# Barriers to Housing for APS Clients: Perspectives From APS Staff and Housing Providers

**DOI:** 10.1093/geroni/igaf122.3135

**Published:** 2025-12-31

**Authors:** Samantha Tuft, Courtney Reynolds, Mia Canzone

**Affiliations:** Benjamin Rose, Cleveland, Ohio, United States; Benjamin Rose, Cleveland, Ohio, United States; Benjamin Rose, Cleveland, Ohio, United States

## Abstract

Oklahoma Adult Protective Services (APS) relies on a patchwork of housing providers, including traditional shelters and nursing homes, to provide clients with emergency housing. To better address its clientele’s needs, APS is partnering with Benjamin Rose to develop an innovative, evidence-based shelter program for APS clients in Oklahoma. To develop the program, a needs assessment was conducted through six focus groups—three with APS staff (n = 20) and three with housing providers (n = 21)—via Microsoft Teams. Thematic analysis revealed significant barriers to housing access, including affordability, lack of available resources, and pets. A common theme was the lack of affordable housing, with one participant noting, “As living costs have increased and those fixed incomes have not, people are shuffling… Do I pay my rent or do I pay for food or do I pay for my medication?” A lack of available resources were also highlighted, as one participant stated, “As soon as you have a list of all the resources in your area, it’s out of date…” Participants also emphasized pets, with one participant sharing, “That’s their best friend, their buddy. Sometimes it’s the reason to continue to live…” These findings underscore the need for safe and affordable housing options (including for those with pets), supportive services, and enhanced collaboration between APS and housing providers. The discussion will address these barriers and offer recommendations to improve housing and service coordination for adults experiencing abuse, neglect and/or exploitation.

